# Discovery of a Novel Species of Trichomonasvirus in the Human Parasite *Trichomonas vaginalis* Using Transcriptome Mining

**DOI:** 10.3390/v14030548

**Published:** 2022-03-06

**Authors:** Austin R. Manny, Carrie A. Hetzel, Arshan Mizani, Max L. Nibert

**Affiliations:** 1 Department of Microbiology, Blavatnik Institute, Harvard Medical School, Boston, MA 02115, USA; austinmanny@g.harvard.edu (A.R.M.); carriehetzel@g.harvard.edu (C.A.H.); arshan@bu.edu (A.M.); 2 Program in Virology, Division of Medical Sciences, Graduate School of Arts & Sciences, Harvard University, Cambridge, MA 02138, USA; 3 Department of Biology, Boston University, Boston, MA 02215, USA

**Keywords:** dsRNA virus, protozoan virus, *Totiviridae*, transcriptome mining, trichomonasvirus, virus discovery

## Abstract

*Trichomonas vaginalis* is the most common non-viral cause of sexually transmitted infections globally. Infection by this protozoan parasite results in the clinical syndrome trichomoniasis, which manifests as an inflammatory disease with acute and chronic consequences. Half or more isolates of this parasite are themselves infected with one or more dsRNA viruses that can exacerbate the inflammatory syndrome. At least four distinct viruses have been identified in *T. vaginalis* to date, constituting species *Trichomonas vaginalis virus 1* through *Trichomonas vaginalis virus 4* in genus *Trichomonasvirus*. Despite the global prevalence of these viruses, few complete coding sequences have been reported. We conducted viral sequence mining in publicly available transcriptomes across 60 RNA-Seq accessions representing at least 13 distinct *T. vaginalis* isolates. The results led to sequence assemblies for 27 novel trichomonasvirus strains across all four recognized species. Using a strategy of de novo sequence assembly followed by taxonomic classification, we additionally discovered six strains of a newly identified fifth species, for which we propose the name *Trichomonas vaginalis virus 5*, also in genus *Trichomonasvirus*. These additional strains exhibit high sequence identity to each other, but low sequence identity to strains of the other four species. Phylogenetic analyses corroborate the species-level designations. These results substantially increase the number of trichomonasvirus genome sequences and demonstrate the utility of mining publicly available transcriptomes for virus discovery in a critical human pathogen.

## 1. Introduction

Trichomonas vaginalis viruses (TVVs) are monosegmented (i.e., nonsegmented) dsRNA viruses that infect the parasitic protozoan *Trichomonas vaginalis* [1]. They constitute genus *Trichomonasvirus* in family *Totiviridae* [1] and are related to monosegmented dsRNA viruses that infect some other parasitic protozoa, namely, viruses that infect *Leishmania* species and constitute genus *Leishmaniavirus* in family *Totiviridae* [2] and viruses that infect *Eimeria* species and constitute proposed genus *Eimeriavirus* in family *Totiviridae* [3]. Monosegmented dsRNA viruses that infect *Giardia* species and constitute genus *Giardiavirus* (currently classified in family *Totiviridae*, although possibly destined for separation) are more distantly related to trichomonasviruses [4]. TVVs encode two proteins: the viral capsid protein (CP) and a fusion protein, comprising both the CP and the viral RNA-dependent RNA polymerase (RdRp), that results from programmed ribosomal frameshifting [1].

The prevalence of *T. vaginalis* as a sexually transmitted human pathogen is a major reason for interest in trichomonasviruses. *T. vaginalis* is an extracellular parasite that attaches to epithelial cells of the genitourinary tract of both women and men and is the causative agent of trichomoniasis, the most common non-viral sexually transmitted infection worldwide. Trichomoniasis is associated with perturbed vaginal microbiota, premature delivery, low birth weight, infertility, and the increased transmission and acquisition of other infectious agents including HIV and HPV [5]. Currently, the antimicrobial drug metronidazole is used to treat trichomoniasis, but drug resistance is rising, indicating a need for alternative therapies [6]. Trichomonasviruses are thought to increase the virulence of *T. vaginalis* by increasing the degree of inflammation during infection. Cervicovaginal epithelial cells sense trichomonasviruses via the recognition of dsRNA by TLR3, which triggers a proinflammatory response through the NF-κB and IRF3 pathways [7].

A somewhat curious aspect of trichomonasviruses is that they comprise strains of at least four species (*Trichomonas vaginalis virus 1* through *Trichomonas vaginalis virus 4*, abbreviated to TVV1 through TVV4 in strain names) [8,9,10,11] and that many *T. vaginalis* isolates are concurrently co-infected with different combinations of these species, including some isolates with all four [10,12]. This finding raises the question of the relative roles played by each species and how each may affect the virulence of *T. vaginalis*. It also presents the possibility of biologically relevant interactions among the species, which may give rise to differential effects on the *T. vaginalis* host, and ultimately, the human superhost. The antiviral response described above, for example, has been demonstrated in response to TVV1, but not yet for any of the other species in the absence of TVV1, to the best of our knowledge.

RNA-Seq transcriptome data deposited in public databases, such as sequence reads in the Sequence Read Archive (SRA) database and transcript assemblies in the Transcriptome Shotgun Assembly (TSA) database, both maintained at the National Center for Biotechnology Information (NCBI; Bethesda, MD, USA), are proving a boon for RNA virus discovery [13]. For the current study, we decided to screen these databases to discover novel trichomonasvirus strains. We found that the SRA database in particular contains sequence reads from a number of different transcriptome studies of *T. vaginalis*. From these SRA datasets, we were then able to assemble complete, nearly complete, or partial coding sequences for 27 novel trichomonasvirus strains across all four recognized species. We supplemented this work by determining the complete coding sequences for two other novel strains by de novo sequencing. Notably, we also implemented de novo assembly methods and sensitive homology searches of distantly related sequences to discover six strains of a fifth trichomonasvirus species. We propose the name *Trichomonas vaginalis virus 5* for this newly identified species, abbreviated to TVV5 in strain names. Comparisons of these TVV sequences enhance our understanding of several basic features of these viruses.

## 2. Materials and Methods

### 2.1. Analyses of Public Transcriptome Data

RNA-Seq transcriptome datasets in the SRA database at NCBI were screened for TVV-matching sequence reads. This analysis was carried out with a locally implemented BLAST [14] instance to retrieve hits, followed by a deduplication step to exclude erroneous reads cross-mapping to other trichomonasvirus species. Discontiguous megablast (‘stand_alone_blast.sh’) was first run with multiple species-specific queries against the respective SRA datasets using an e-value threshold of 1 × 10^−9^. The accessions of these queries obtained from NCBI GenBank are as follows: TVV1: U08999.1, DQ270032.1, HQ607516.1, HQ607513.1, HQ607517.1, JF436869.1; TVV2: NC_003873.1, HQ607514.1, HQ607518.1, HQ607524.1, JF436870.1, JF436871.1; TVV3: NC_004034.1, HQ607515.1, HQ607519.1, HQ607525.1; TVV4 HQ607522.1, HQ607520.1, HQ607526.1. Blastn (‘cleanup_blast.sh’) was subsequently run on those hits against a local blast database containing 80 TVV sequences retrieved from GenBank, using an e-value threshold of 10. This confirmed how many of the initial hits indeed best matched the queried trichomonasvirus species. Table 1 shows these results for the number of TVV-matching reads for each respective species in each dataset. In addition to this map-to-reference strategy, a de novo assembly approach was used for the discovery of divergent TVV sequences in the SRA datasets. Identification of the initial TVV5 strain was achieved using rnaSPAdes (v1.13.0) [15] to assemble sequences. These de novo assemblies were queried against the NCBI nonredundant protein (nr) database using DIAMOND (v0.9.21) [16] in blastx mode. The DIAMOND parameter ‘--top 1’ was used to force the lowest common ancestor (LCA) algorithm to consider the 99th percentile of the reference sequences to determine the taxonomic origin of each assembly. A custom Python (v3.7.3) script (‘diamondToTaxonomy.py’) was used to convert NCBI taxonomy IDs to full taxonomic lineages using the JGI-DOE taxonomy server (taxonomy.jgi.doe.gov). Sequence assemblies assigned to family *Totiviridae* were selected for further analysis. These viral assemblies were refined by mapping the reads assigned by BLAST to generate a 50% consensus sequence. This refinement step was performed using a mapping script (‘refine_contigs.sh’) that implemented BWA-MEM (v0.7.17) [17], SAMtools (v1.10) [18], and BCFtools (v1.10.2) [19]. Finalized reads for each novel TVV strain were then assembled into coding-complete, nearly coding-complete, or partial sequences (Supplementary File S1) using CAP3 (v02/10/15; parameters ‘-o 21 -p 66 -s 300 -z 2’) [20] and CLC Genomics Workbench (v8.0.1; QIAGEN, Redwood City, CA, USA). Normalized coverage values, expressed as RPKM (Supplementary Table S1), were calculated for each assembly. Sequencing depth was also calculated by mapping each set of TVV-matching reads to a reference strain for that species using BWA-MEM. The SAMtools ‘depth’ function was used to determine the number of mapped reads per nucleotide (nt) position, and a median value was calculated for each virus assembly (Supplementary Table S2) using the ‘median’ function in the statistical programming language R (v4.1.2) [21]. Plots presented throughout this study were developed in R using ggplot2 (v3.3.5) [22] with extensive use of the tidyverse (v1.3.1) framework [23].

### 2.2. Phylogenetic Tree Construction

**Figure 1:**
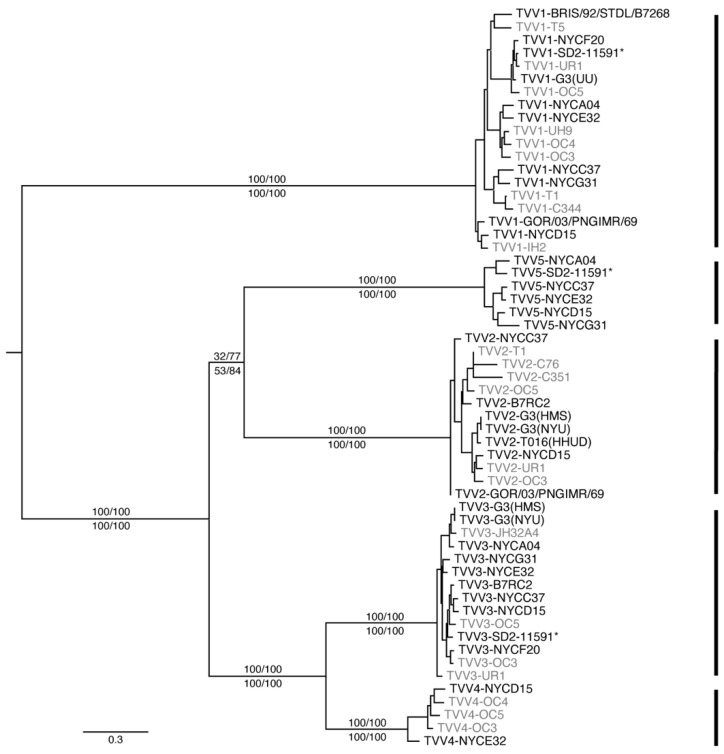
Maximum-likelihood phylogenetic tree of TVV1 through TVV5 strains. CP/RdRp aa sequences were deduced from the new TVV assemblies presented in this study (labeled in black) as well as from reference TVV genomes retrieved from NCBI GenBank (labeled in gray). Support values for the main branches are shown as percentages; above the branch is the value from standard bootstrapping with/without subsequent transfer analysis, and below the branch is the value from ultrafast bootstrapping with/without subsequent transfer analysis. The tree is rooted at the midpoint. Bars on the right highlight the five trichomonasvirus species. See Table 1 and Supplementary File S2 for explanations of TVV strain names.

Amino acid (aa) sequences for fusion protein CP/RdRp were deduced from newly assembled TVV1 through TVV5 nt sequences and reference TVV1 through TVV4 nt sequences retrieved from NCBI GenBank (Supplementary Files S1 and S2); partial sequences from GenBank were excluded. These sequences were then aligned with MAFFT (v7.490) [24] using the L-INS-i algorithm (Supplementary File S3). Maximum-likelihood phylogenetic trees were built from this alignment using IQ-TREE (v1.6.11) [25] on the Los Alamos National Lab webserver (hiv.lanl.gov). The ‘find best and apply’ option [26] consistently identified JTT+F+I+G4 as the best model. Either standard bootstrapping (Felsenstein; *n* = 100) or ultrafast bootstrapping (UFBoot2 [27]; *n* = 1000) was conducted in consecutive runs of IQ-TREE, and consensus and bootstrap trees were saved from each run. Each set of trees was then used for transfer analysis by BOOSTER (v0.1.9) [28] on the Pasteur Institute webserver (booster.pasteur.fr). As expected, an essentially identical consensus tree was obtained from each analysis: identical in terms of branch topologies and branch lengths limited to two or three significant digits but differing in the support values for some branches. For optimized presentation, the consensus tree was visualized using FigTree (v1.4.4). Support values from both standard and ultrafast bootstrapping, both without and with subsequent transfer analysis, were provided for the main branches, as described in the Figure 1 legend. For reference, the original Newick tree files for these four different types of bootstrapping are provided as Supplementary Files S4–S7. The same analyses and presentation steps were additionally performed with the nt sequences of these TVV strains (Supplementary Figure S1), rather than with the CP/RdRp aa sequences, as shown in Figure 1.

### 2.3. De Novo Analyses of Isolate G3

For polymerase chain reactions, total RNA was isolated from a sample of *T. vaginalis* isolate G3 obtained directly from the American Type Culture Collection (ATCC; Manassas, VA, USA; accession PRA-98) and not subjected to laboratory culturing. cDNA was synthesized using SuperScript III reverse transcriptase (Invitrogen, Carlsbad, CA, USA) with random hexamer primers. A 50 µL polymerase chain reaction was carried out for each virus (TVV1, TVV2, TVV3, TVV4, and TVV5) using Taq polymerase (New England Biolabs, Ipswich, MA, USA) for 35 PCR cycles. PCR products were visualized on a 1% agarose gel alongside 1 Kb Plus DNA Ladder (Invitrogen). Primer sequences are listed in Supplementary Table S3.

For high-throughput sequencing, isolate G3 from ATCC was minimally cultured in Diamond’s modified Medium [29] for three passages at late-exponential phase. Total RNA was isolated using TRIzol. Contaminating ssRNA was depleted with a 2M LiCl incubation at −20°C overnight. Final enrichment was achieved through nuclease digestion using DNase I and S1 nucleases (Promega Corporation, Madison, WI, USA). Enriched dsRNA was shipped overnight on dry ice to Quick Biology (Pasadena, CA, USA) for sequencing. The RNA-Seq library was prepared according to the KAPA KK8540 RNA HyperPrep kit with 201–300 bp insert size (KAPA Biosystems, Wilmington, MA, USA) using 25–50 ng of total dsRNA as input. Final library quality and quantity were analyzed with the Agilent (Santa Clara, CA, USA) Bioanalyzer 2100 and Invitrogen Qubit 3.0 Fluorometer. Paired-end 150 bp reads were sequenced on an Illumina (San Diego, CA, USA) HiSeq 4000. Sequences were demultiplexed to remove barcodes at the sequencing facility.

For bioinformatic analysis, adapters and other technical sequences were trimmed from the preceding paired-end reads using TrimGalore (v0.6.3) (https://github.com/FelixKrueger/TrimGalore; accessed on 13 July 2019) with the following parameters: ‘paired, stringency=5, quality=20’. Bacteriophage ΦX174 positive-control spike-in reads were depleted using BWA-MEM with default parameters. Reads were assigned to their respective trichomonasvirus species using the BLAST workflow above (i.e., initial mapping with discontiguous megablast followed by a clean-up step with blastn). After discarding the reads, species-specific reads were de novo assembled into a draft genome using CAP3 (parameters: ‘-o 21 -p 66 -s 300 -z 2’). In addition to this map-to-reference approach, the de novo assembly strategy described above was also employed. Both approaches converged on the same result, yielding coding-complete sequences from *T. vaginalis* isolate G3 for both TVV2 and TVV3.

## 3. Results

### 3.1. Screening for Trichomonasvirus Sequences in Public Transcriptomes

At the time of this study, the SRA database at NCBI contained 60 individual accessions from RNA-Seq transcriptome studies of *T. vaginalis* deposited under five BioProjects from four institutions in the United States, Germany, and Korea [30,31,32,33]. These accessions encompass sequence reads from at least 13 distinct *T. vaginalis* isolates. In many cases, the same isolate was represented by several different accessions, which we combined to yield the 16 distinguishable SRA datasets that we subjected to analysis, as listed in Table 1. We first screened these datasets for the presence of reads matching any of the four recognized species in genus *Trichomonasvirus* [1,8,9,10,11]. Briefly, we applied discontiguous megablast at NCBI for performing the database searches, using three to six previously reported nt sequences for each of the four species as queries. Multiple queries were used for each species in an effort to increase the numbers of identified hits from divergent strains of each species that might have been present in these *T. vaginalis* isolates. Results of the screens are summarized in Table 1, with numbers that reflect apparently novel TVV strains shown in bold.

Sequence reads matching all four trichomonasvirus species were found, often in large numbers. Only two of the 16 SRA datasets that we distinguished for screening were concluded to be negative for all four species (0 to 13 hits per species): the datasets for *T. vaginalis* isolate T016 from BioProject PRJNA352855 and *T. vaginalis* isolate NYCB20 from BioProject PRJNA280779. The other 14 datasets were concluded to be positive for at least one species each (67 to 26,085 hits per species): ten for TVV1, seven for TVV2 (including two from the same isolate and institution), nine for TVV3, and two for TVV4. Broken down by *T. vaginalis* isolate, isolates G3 (as reported from BioProject PRJNA345042, University of Utah (UU)) and BRIS/92/STDL/B7268 were positive for TVV1 only; isolate T016 (as reported from BioProjects PRJNA176299 and PRJNA236636, Heinrich Heine University Düsseldorf (HHUD)) was positive for TVV2 only; isolate GOR/03/PNGIMR/69 was positive for TVV1 and TVV2; isolates NYCA04, NYCF20, NYCG31, and SD2 11591* (asterisk a part of isolate name) were positive for TVV1 and TVV3; isolates G3 (as reported from BioProject PRJNA345042, New York University (NYU)) and B7RC2 were positive for TVV2 and TVV3; isolate NYCC37 was positive for TVV1, TVV2, and TVV3; isolate NYCE32 was positive for TVV1, TVV3, and TVV4; and isolate NYCD15 was positive for all four species (Table 1). In total, the screening results suggest the identification of 27 novel trichomonasvirus strains, as additionally examined below.

### 3.2. Assembly of Novel Trichomonasvirus Genome Sequences

Having complete coding sequences for virus strains is useful for confirming expected features such as open reading frames and ribosomal frameshifting motifs, for identifying conserved features not previously recognized, for identifying phenotypically important sequence variations, and for allowing robust phylogenetic comparisons. We therefore next assembled the TVV-matching reads into complete coding sequences for as many of the 27 novel trichomonasvirus strains as we could. For this, we used the programs CAP3 and CLC Genomics Workbench to assemble the reads into contigs, and we also separately performed de novo assembly of contigs from the SRA datasets, using the program rnaSPAdes, for corroboration of the results. Through this combination of approaches, we were able to generate and confirm complete coding sequences for thirteen novel strains: ten TVV1 (TVV1-G3(UU), TVV1-BRIS/92/STDL/B7268, TVV1-GOR/03/PNGIMR/69, TVV1-NYCA04, TVV1-NYCC37, TVV1-NYCD15, TVV1-NYCE32, TVV1-NYCF20, TVV1-NYCG31, and TVV1-SD2-11591*) and 3 TVV2 (TVV2-T016, TVV2-GOR/03/PNGIMR/69, and TVV2-NYCD15). In addition, we were able to generate and confirm nearly complete coding sequences for four other novel strains: one TVV2 (TVV2-B7RC2; small 3′ truncation) and three TVV3 (TVV3-B7RC2, TVV3-NYCA04, and TVV3-NYCD15; single small gap in each). Lastly, for the remaining ten novel strains suggested by the findings in Table 1 (two TVV2, six TVV3, and two TVV4), we were able to generate and confirm three to six contigs of ≥300 nt in length for each, allowing them also to be included in the subsequent comparisons. RPKM values, using the final full sets of reads used for generating the final assemblies, ranged from 1.2 to 31 for the thirteen coding-complete assemblies (median, 5.2), 0.3 to 2.9 for the four nearly coding-complete assemblies (median, 0.8), and 0.4 to 1.5 for the longest contig from each of the ten partial assemblies (median, 0.6) (Supplementary Table S1). Median sequencing depth values ranged from 10 to 621 for the thirteen coding-complete assemblies (median, 43), 6 to 16 for the four nearly coding-complete assemblies (median, 10), and 3 to 10 for the longest contig from each of the ten partial assemblies (median, 3) (Supplementary Table S1).

### 3.3. Discovery of a Fifth Trichomonasvirus Species

Following our map-to-reference strategy to characterize additional strains of the four recognized trichomonasvirus species, we next devised an approach that could enable discovery of divergent viruses in these same *T. vaginalis* isolates. A de novo assembly approach was implemented, whereby all adapter-trimmed RNA-Seq reads from a given isolate were assembled using De Bruijn graph-based methods. These assemblies were dynamically translated into the protein space and compared with the NBCI nonredundant protein (nr) database using DIAMOND. Assemblies were compared with the top 1% of matching sequences in the reference database and a taxonomic origin was thereby assigned. Assemblies assigned to viral taxa were retrieved and analyzed.

This assembly first approach successfully reconstructed the TVV sequences obtained from the preceding map-to-reference strategy and also enabled a search for more divergent viruses. Accordingly, *T. vaginalis* isolate NYCE32 was found to carry a virus that could be confidently assigned only to genus *Trichomonasvirus*, with no species-level determination. This 5042 bp assembly exceeded the length of any known TVV strain, although it shared the familiar genomic architecture of two large overlapping reading frames of approximately equal length. Querying this assembly using blastn against the NCBI nonredundant nucleotide (nt) database indicated its strongest match to be trichomonasvirus TVV2-UR1. Global pairwise alignments were conducted between the 5042 bp assembly and TVV2-UR1 using Clustal Omega (v1.83) [34], demonstrating only 45% identity between these nt sequences and only 36% identity between their deduced CP/RdRp aa sequences. Different species in the family *Totiviridae* are commonly demarcated by a <50% aa-sequence identity [1], which suggested that isolate NYCE32 harbors a novel trichomonasvirus species.

To test whether other *T. vaginalis* isolates may contain similar viruses to the one in NYCE32, we used the map-to-reference approach with the NYCE32 assembly to identify matching reads in any other dataset. This led us to discover homologous sequences in five other isolates (NYCA04, NYCC37, NYCD15, NYCG31, and SD2-11591*), yielding a total of six additional assemblies of this divergent trichomonasvirus. Two of these assemblies are coding-complete, with a third nearly so (two small gaps); one assembly is partial but encodes a full-length CP; and the remaining two assemblies are partials not fully spanning either viral gene. For the five sequences that cover the predicted CP/RdRp junction, a ribosomal frameshifting site was predicted to involve the heptanucleotide slippery sequence GGGCCCC, which is the same motif as in TVV2. CP/RdRp aa sequences deduced from these six assemblies share >77% pairwise identity with each other, although <50% pairwise identity with any strain of TVV1 through TVV4.

Each of the four recognized trichomonasvirus species represents a distinct monophyletic clade of TVV strains [10]. To examine the relationship of the six divergent assemblies to strains of the recognized species, we constructed maximum-likelihood phylogenetic trees from the aligned CP/RdRp aa sequences of all new TVV assemblies plus reference TVV genomes retrieved from NCBI GenBank (Figure 1). The six divergent assemblies were found to form a monophyletic, discrete clade of their own, with 100% branch support regardless of the different bootstrapping methods we tested. This discrete clade is positioned as sister to the TVV2 clade, consistent with the predicted ribosomal slippery sequence that its members appear to share with TVV2 strains. Given the evidence presented thus far, i.e., a strongly supported discrete clade, high within-clade sequence identity but low sequence identity to TVV1 through TVV4 strains, and a genome length that exceeds that of any previously known TVV strain, we concluded that these six assemblies represent six strains of a novel trichomonasvirus, TVV5, for which we propose the species name *Trichomonas vaginalis virus 5*.

Support values for the branch uniquely shared by the TVV2 and TVV5 clades in Figure 1 are lower than those for the other main branches; thus, we recognize that this specific feature of the tree, i.e., the sister relationship between TVV2 and TVV5, might or might not hold up as other novel TVV strains are added in future analyses. It is notable, however, that maximum-likelihood trees we constructed from the nt sequences of these same TVV strains, rather than from their CP/RdRp aa sequences as for Figure 1, show discrete TVV2 and TVV5 clades that are again positioned as sisters and have even somewhat stronger support values for their uniquely shared branch (Supplementary Figure S1).

### 3.4. Reexamination of T. vaginalis Isolate G3

Our results for the presence of TVV sequences in the analyzed SRA datasets (Table 1) include inconsistent findings for *T. vaginalis* isolate G3. In particular, G3 from the University of Utah (BioProject PRJNA345042) is positive only for TVV1, whereas G3 from New York University (BioProject PRJNA280779) is positive instead for TVV2 and TVV3. Isolate G3 can be purchased from ATCC and is also the reference isolate for which the *T. vaginalis* whole-genome sequence draft has been reported [35]; therefore, we newly acquired and examined this isolate for the presence of trichomonasviruses.

For detecting virus strains that may be carried by isolate G3, we first designed primer pairs based on the TVV1-G3(UU), TVV2-G3(NYU), and TVV3-G3(NYU) sequences described above. Primers for TVV4 and TVV5 were designed against conserved regions of available sequences. RNA was then extracted directly from the cells of *T. vaginalis* isolate G3 that were present in the original sample sent from ATCC, followed by RT-PCR using the respective primer pairs and assay by agarose gel electrophoresis. In this manner, we consistently failed to obtain an amplicon of the expected size using the primer pair based on TVV1-G3(UU) but succeeded in obtaining amplicons of the expected sizes using the primer pairs based on TVV2-G3(NYU) and TVV3-G3(NYU). Moreover, when the latter amplicons were subjected to Sanger sequencing, we found them to be 100% identical to the respective region of TVV2-G3(NYU) (amplicon length excluding primers, 715 nt) and 99.6% identical to the respective region of TVV3-G3(NYU) (amplicon length excluding primers, 798 nt). As expected, reactions for TVV4 and TVV5 did not yield amplicons. Based on these results, we conclude that the TVV results obtained for *T. vaginalis* isolate G3 reported from New York University are representative of those for G3 isolates currently available from ATCC.

The assembled sequences for TVV2-G3(NYU) and TVV3-G3(NYU) from BioProject PRJNA280779 are not coding-complete; therefore, we also performed an RNA-Seq analysis of *T. vaginalis* isolate G3 that we acquired from ATCC, in an effort to complete the coding sequences of these TVV strains. Following the enrichment of dsRNA from this isolate after limited passage in culture in our laboratory, the sample was submitted for RNA-Seq analysis by a commercial vendor. Results revealed the presence of many sequence reads matching TVV2 and TVV3, but not TVV1, TVV4, or TVV5 (Table 1), consistent with the results from BioProject PRJNA280779 described above, as well as with our RT-PCR results. Additionally, reads sufficient in number and coverage were obtained to assemble complete coding sequences for both TVV2-G3(HMS) and TVV3-G3(HMS) (HMS reflecting that this study was performed at Harvard Medical School; see Supplementary Tables S1 and S2 for assembly statistics). These new assemblies exhibit 100% sequence identity with the amplicons for portions of these viruses described above and 99.7% and 99.3% identity, respectively, with the SRA-based partial assemblies for TVV2-G3(NYU) and TVV3-G3(NYU).

### 3.5. Comparisons of Trichomonasvirus Sequences

Untranslated regions (UTRs) at the termini of viral genomes are important sites for replication and/or packaging. Previous reports [10] have found conserved UTR elements across the four recognized trichomonasvirus species. UTRs routinely exhibit more sequence flexibility than protein-coding regions, as insertion–deletion (indel) events in the latter can result in deleterious frameshifts and yield defective viral proteins. However, as noted above, UTRs can play vital functional roles too, which can constrain genetic variation in these regions. All TVVs exhibit long 5′ UTRs that could contain functional elements such as internal ribosomal entry sites.

**Figure 2:**
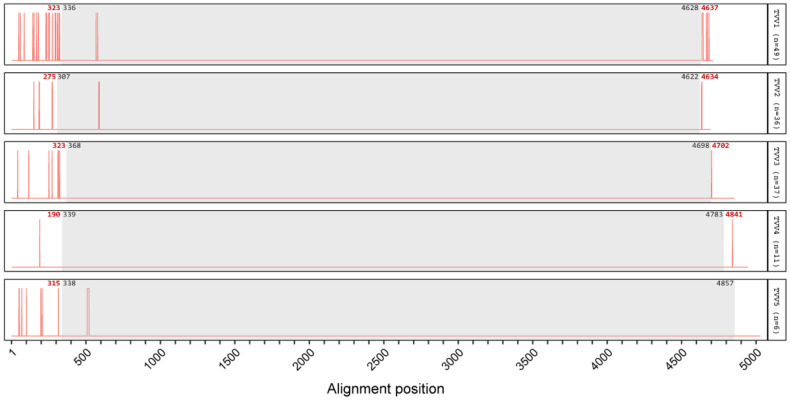
Gap plots showing all indels across the aligned nt sequences of TVV1 through TVV5 strains. Indels are concentrated in the 5′ and 3′ UTRs, although gapless regions also found within the UTRs suggest conserved functional elements. For each trichomonasvirus species, new assemblies were combined with all coding-complete and partial sequences from NCBI GenBank and aligned using MAFFT L-INS-i. The unsequenced ends of partial sequences in the multiple sequence alignment were masked to prevent bias from missing residues. The alignment was analyzed with a custom R script. Gray boxes denote the CDS for the CP/RdRp of each species. Gap positions are indicated by red bars. Red numbers indicate the gap position nearest each CDS boundary; black numbers indicate the CDS boundary positions.

**Figure 3:**
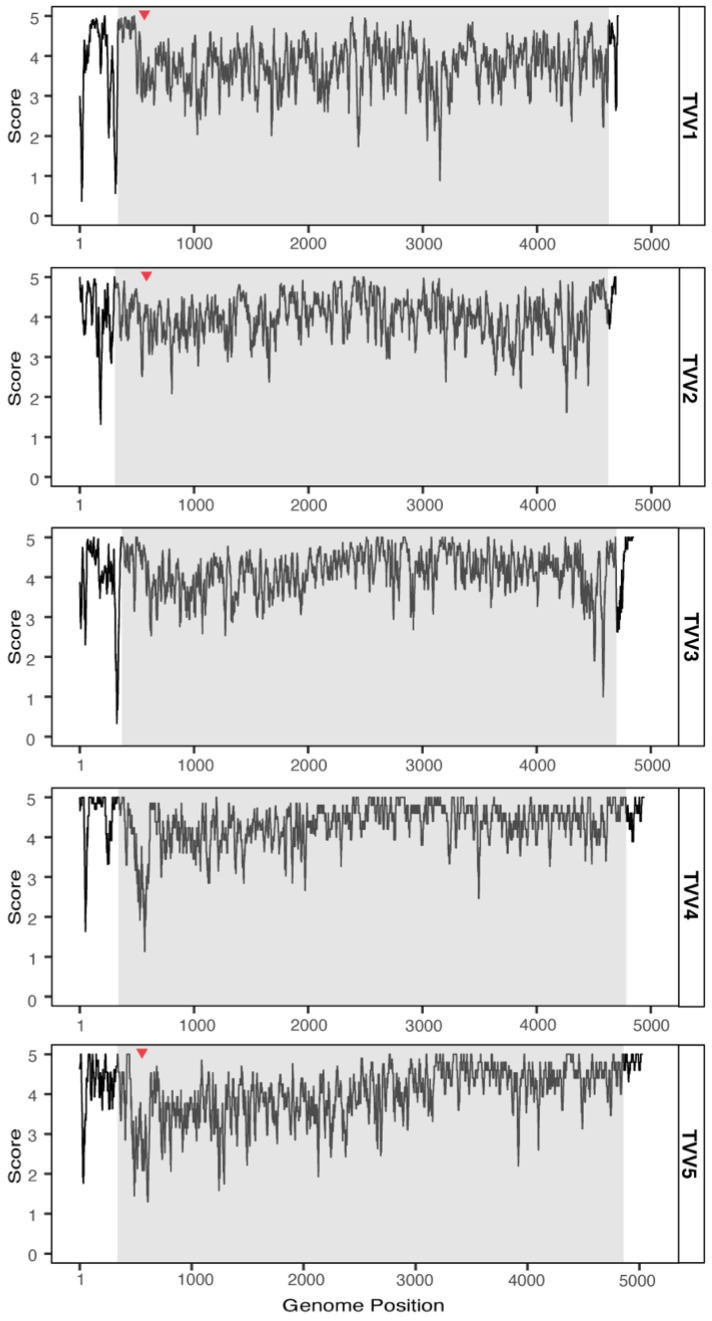
Conservation plots for nt sequences of TVV1 through TVV5 strains. For each trichomonasvirus species, complete and partial coding sequences were retrieved from NCBI GenBank and combined with new assemblies that were coding-complete or nearly so. A sliding window of 15 nt was chosen for smoothing. The EDNAFULL substitution matrix was used, in which a score of 5 denotes perfect identity at a given position. Gray boxes denote the CDS for the CP/RdRp of each species. Red triangles denote positions of any indels within the CDS (also see Figure 2).

To search for functional elements in the UTRs, all new TVV assemblies were compared with the currently available TVV sequences in NCBI GenBank. Per species, these sequences were aligned and any gaps (corresponding to indels between strains) were plotted (Figure 2). Most gaps are located in the UTRs, especially the 5′ UTR. On the other hand, there are distinct regions in the 5′ UTR that lack gaps. As TVV1 is represented by the most sequences, this pattern can be most clearly seen for it: two long gap-free zones where no nucleotides have been inserted or deleted in the available sequences. To investigate whether genetic variation at these nt positions is constrained, conservation plots were also generated for each trichomonasvirus species (Figure 3). The conservation plot for TVV1 shows two peaks of high sequence identity at the two gapless regions. The beginning of the CP gene is also highly conserved. This pattern of long 5′ UTRs including distinct zones with constrained sequence variation and no indels points to a functional role for the 5′ UTR of TVVs and allows for the possibility of an internal ribosomal entry site in all five trichomonasvirus species.

Indels were found to be largely excluded from the protein coding sequences (CDSs) of TVV genomes. No strains of TVV3 or TVV4 possess gaps within the CDS; all indels are in either the 5′ or 3′ UTR. One strain of TVV5 (TVV5-NYCA04) appears to possess a 12 bp deletion in the CDS, near the beginning of the CP gene and corresponding to a skip of 4 aa. TVV1 and TVV2 were found to each have two GenBank sequences with apparent insertions in the CDS. For TVV1, both sequences are for small amplicons from the study of an Iranian patient cohort [36]. TVV1-SH8 (GenBank accession AB701566.1) is a 149 bp amplicon sequence and contains an inserted guanosine residue at position 568 relative to the whole genome alignment with all other TVV1 strains. TVV1-SH4 (GenBank accession AB701562.1) is a 142 bp amplicon sequence with an inserted adenosine residue at position 579. In both cases, the inserted residue results in a frameshift that would yield a truncated CP with a divergent C-terminus. Removal of the 1 nt insertion in each of these cases restores the resulting CP sequence to match that in other known TVV1 sequences. For TVV2, both sequences with apparent insertions in the CDS are from the study of a Cuban patient cohort [37]. TVV2 strains C76 and C351 (GenBank accessions JF436870.1 and JF436871.1) each contain a multiple nt insertion near the beginning of the CP gene. TVV2-C76 possesses the insertion AAGAAA at positions 585–590, and TVV2-C351 possesses the insertion TAA at positions 588–590. These insertions maintain the coding frame and would result in the respective introduction of two or one extra aa into the CP. The indels identified within the CDS of these few TVV1, TVV2, or TVV5 strains might or might not reflect sequencing or assembly artifacts.

TVV1 genomes were found to possess a conserved CUUUUUGCAC element in the 5′ UTR of full-length sequences. The sole exception is TVV1-NYCC37, which has an extra uracil residue resulting in CUUUUUUGCAC. This uracil-rich element is found in all full-length TVV1 sequences but not in any strains of TVV2, TVV3, TVV4, or TVV5. The 3′ UTRs of each species demonstrate fairly strong sequence conservation within a species, with little similarity across species. These species-specific UTR elements could play a role in the segregation of viral components during prevalent co-infections with multiple trichomonasviruses in a single protozoan host.

Interestingly, our newly assembled TVV1 sequences are all coding-complete, have 5′ termini that match or exceed in length those of previously reported TVV1 genomes, and have 3′ termini that extend within 20 nt of reference TVV1 genomes. Three of the seven TVV2 sequences newly assembled from SRA datasets are coding-complete, with a fourth nearly so. None of the nine TVV3 sequences newly assembled from SRA datasets are coding-complete, but three are nearly so. Neither of the two newly assembled TVV4 sequences is coding-complete or nearly so. Lastly, two of the six newly assembled TVV5 sequences are coding-complete, with a third nearly so. Genome completeness was found to be correlated with coverage depth (*p* = 0.001; Supplementary Figure S2). To evaluate whether the number of mapped reads is related to trichomonasvirus species, a nonparametric Kruskal–Wallis test was conducted to evaluate whether each species had equivalent numbers of mapped reads per sample (normalized as RPKM values). RPKM values were found to vary significantly across species (*p* = 0.029), presenting the possibility of different levels of each TVV within a host protozoan (Supplementary Figure S3). To evaluate sequence conservation across trichomonasvirus species, CP/RdRp aa sequences were deduced from newly assembled sequences and globally aligned per species using Clustal Omega. Pairwise identity matrices for each trichomonasvirus species are provided in Supplementary Tables S4–S8, which show that the analyzed strains share ≥77.9% pairwise identity within each species.

All TVV1 genomes assembled in this study are coding-complete, which allowed for the most detailed analysis of a trichomonasvirus species. Almost all TVV1 CP/RdRp sequences are 1430 aa in length, based on predicted initiation at the first in-frame AUG codon. However, both TVV1-UR1-1 and SD2-11591* contain an MGIP N-terminal extension, bringing their apparent lengths to 1434 aa. All TVV1 sequences share a triple serine at their C-termini, except for TVV1-NYCC37, which has STS. Average pairwise identity across the TVV1 CP/RdRp aa sequences is 85.9%. Scanning across the CP/RdRp sequence with a window of 10 aa reveals a single area of the genome with <50% conservation. This region occurs halfway through the CP/RdRp aa sequences, centered on position 701 (705 for TVV1-UR1-1 and SD2-11591*). Interestingly, absolute conservation is observed 20 aa upstream, the site of the ribosomal frameshift.

For TVV2, average pairwise identity for the CP/RdRp aa sequences is 88.3%. The CP/RdRp sequences deduced from coding-complete TVV2 genomes are 1438 aa in length. The N-terminal motif MASTL is found in all these sequences, except in TVV2-T016(HHUD), where it is MAATL. The C-termini of all full-length TVV2 fusion proteins end with PVYV. For TVV3, average pairwise identity for the CP/RdRp aa sequences is 92.1%. The N-termini of all TVV3 sequences that extend to this region begin with MSAPEPLNTEVR, and the C-termini of all TVV3 fusion proteins that extend to this region end with GHGLRSG. Analysis of the TVV4 CP/RdRp aa sequences was impeded by the fact that no TVV4 genomes that are coding-complete or nearly so could be newly assembled from the SRA datasets. The TVV4-NYCD15 and TVV4-NYCE32 assemblies contain partial CP-coding sequences, which have a pairwise identity score of 90.3%. These sequences were aligned to the CP-coding sequences of the three TVV4 genomes in GenBank: TVV4-OC3, TVV4-OC4, and TVV4-OC5. TVV4-NYCD15 shares the N-terminal motif MSAI with TVV4-OC3 through TVV-OC5. Neither new TVV4 assembly reaches the CP C-terminus.

TVV5 CP/RdRp proves to be the largest of all trichomonasvirus proteins. The three assemblies that are coding-complete or nearly so encode a fusion protein of 1506 aa. Examination of all six TVV5 strains shows a largely conserved N-terminus. The exception is TVV5-NYCA04, which possesses a guanosine at genome position 283 (instead of the adenosine found in all other sequences with coverage at that position), yielding a different proposed start codon. Along with a nearby single nt deletion, this results in an N-terminal extension of 18 aa in the CP and CP/RdRp of this strain. Three TVV5 sequences maintain coverage through the CP/RdRp C-terminus, which ends in PAVPIAT in all three.

Programmed ribosomal frameshifts are instrumental in TVV biology, giving rise to the catalytic CP/RdRp fusion protein. All strains sequenced in this report demonstrate absolute conservation of the known or predicted heptanucleotide slippery sequence in each respective species: TVV1, CCUUUUU; TVV2/TVV5, GGGCCCC; and TVV3/TVV4, GGGCCCU. No variation in these motifs was observed for any strain of any species. A common observation across all TVV species is that although the aa sequences deduced from the region surrounding the ribosomal frameshift are strictly conserved, 10–30 aa downstream is a region of extreme divergence. This observation is described above for TVV1. For another example, the TVV2 ribosomal frameshift site represents position 699 in the deduced aa sequence, and positions 693–702 are perfectly conserved across all strains. However, shortly downstream is a region of low conservation, dropping below 50% at positions 737–738, after which higher sequence identity (80–95%) is restored. The evolutionary constraint of the ribosomal frameshift might cause higher diversifying selection pressure in the neighboring genomic space, or perhaps this region of aa sequence shortly downstream of the frameshifting site is less subject to structural constraints, possibly representing a “hinge” between CP and RdRp portions of the fusion protein.

**Figure 4:**
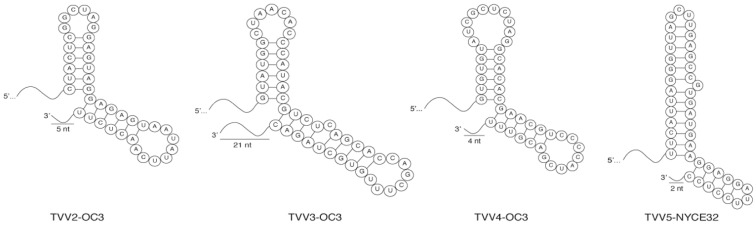
Double stem–loop structures near the coding-strand 3’ termini of TVV2 through TVV5 strains. Secondary-structure predictions identified this conserved feature, shown here for representative strains TVV2-OC3, TVV3-OC3, TVV4-OC3, and TVV5-NYCE32. This feature extends to within 21 nt of the coding-strand 3’ terminus of each virus and consists of two adjoining stem–loops with no intervening nt residues.

To evaluate conserved, terminal RNA structures across trichomonasviruses, secondary-structure predictions were performed for strains of all five species. Specifically, the first and last 100 nt from all TVV strains whose available sequences closely approach one or both expected termini of the TVV genome were analyzed for RNA secondary structures at the 5′ or 3′ end of their coding strands using RNAfold (v2.4.18) [38]. As expected from previous reports [10], a long stem–loop structure was again predicted at the 5′ terminus of each analyzed sequence, regardless of species and including for TVV5 strains. In addition, strains of TVV2, TVV3, TVV4, and TVV5 were predicted to possess a double stem–loop structure at the 3’ terminus of each analyzed sequence (Figure 4). This structure consists of two adjoining stem–loops with no intervening nt residues. These structures extend to within 5 nt of the expected 3′ terminus in TVV2, TVV3, and TVV5 strains, and to within 21 nt of the expected 3′ terminus in TVV4 strains. This conserved RNA feature could play a role in packaging the genome into virions and/or recognition by the viral RdRp.

## 4. Discussion

Discovery of a fifth species in genus *Trichomonasvirus* emphasizes the utility of mining viral sequences from publicly available transcriptomes. A map-reads-to-reference strategy was initially useful for characterizing novel strains of the four recognized trichomonasvirus species. An orthogonal ‘assemble-first, classify-later’ approach then allowed us to uncover the newly identified fifth species infecting *T. vaginalis*. These complimentary methods enable a robust exploration of sequence data while incorporating the flexibility needed for finding new viruses. Each new TVV assembly held up to rigorous scrutiny, corroborated in all cases by both reference-based and de novo assembly methods. Supplementary Table S1 shows the normalized read count per virus assembly presented in this report, expressed as RPKM values. Most assemblies have an RPKM of <5. Relatedly, most of these assemblies have less than 20x coverage (Supplementary Table S2). These values are high enough to allow the assembly of viral sequences and yielded the assembly of several coding-complete TVV genomes, as presented in this report. However, the fact that these values are relatively low might also explain the number of partial assemblies we obtained. For example, for the two newly assembled TVV4 sequences, both of which are partials, each RPKM was <1 and the median coverage was <10x. Although viral sequence mining was demonstrably successful using these untargeted datasets, methods for enriching viral genomes or transcripts in RNA-Seq libraries may be considered for the reliable determination of more coding-complete viral genomes.

Co-infection of many *T. vaginalis* isolates with strains of two or more trichomonasvirus species, as seen previously [10] and again in this study, raises many interesting questions, including whether the different species might be subject to recombination. Although some other RNA viruses (e.g., coronaviruses) exhibit frequent detectable recombination events [39,40], the extent of recombination among trichomonasviruses appears to be low. Pairwise alignment of representative strains of the five species failed to reveal any obvious chimeric breakpoints indicative of recombination events. Furthermore, detailed analysis with the DualBrothers package (v1.1.5) [41] failed to detect recombination between TVV strains. This is perhaps not surprising, because most dsRNA viruses are thought to replicate their individual genomes only within the confines of their protein capsids, in which their RdRp or CP/RdRp molecules are also packaged [42]. This capsid barrier may sequester the replicating RNAs from the potentially distinct RNAs inside other capsids, reducing the chance of interspecies recombination and replication of chimeric molecules. Moreover, the subcellular localizations of different trichomonasviruses within a single protozoan remain unclear. It is possible that during viral co-infections of a shared protozoan cell, distinct TVV strains are additionally secluded from one another in some manner, further reducing the chance of interspecies recombination.

Goodman et al. [10] reported a conserved, short 5′-terminal region of sequences (36 or 37 nt) in strains TVV1-UH9, TVV1-UR1, TVV1-OC3, TVV1-OC4, and TVV1-OC5 (GenBank accessions HQ607516.1, HQ607513.1, HQ607517.1, HQ607521.1, and HQ607523.1), which was missing from previously reported sequences for strains TVV1-T1, TVV1-T5, and TVV1-IH2 (GenBank accessions U08999.1, U57898.1, and DQ270032.1) [11,43,44] and is also missing from the subsequently reported sequence for strain TVV1-C344 (GenBank accession JF436869.1) [34]. This short sequence extension is notable because it allows the formation of a long 5′-terminal stem–loop structure, which seems likely to be involved in RNA stability and/or other functions in TVV1 replication. All of the new TVV1 sequences reported here extend into this conserved 5′-terminal region, providing further evidence that the sequences for TVV1-T1, TVV1-T5, TVV1-IH2, and TVV1-C344 are likely truncated at their 5′ ends. The fact that the new TVV1 sequences appear to be less but still partially truncated themselves is not surprising, given that RNA-Seq-derived transcript assemblies, in our experience, are often missing a few residues at each end of respective transcripts.

Strains of all five trichomonasvirus species have long 5′ UTRs, >280 nt each, as well as numerous AUG codons preceding the first in-frame AUG codon in the CP gene [1]. These features, also evident in the novel strains reported here, suggest to us that at least some of these 5′-UTR sequences contribute to forming an internal ribosome entry site/structure important for viral translation, as shown or suggested for several other members of family *Totiviridae* or related dsRNA viruses [45,46]. The increased number of complete coding sequences now available for alignment as a consequence of the current study additionally reveals that indels between TVV strains are found in the 5′ UTR of each trichomonasvirus species, as shown in Figure 2. These indels may mark sequence locations that are not directly involved in essential functions at the RNA level or in forming essential RNA structures. Indels between strains have also been found in the 3′ UTRs of four trichomonasvirus species but are generally not found in the long CDS that occupies most of each genome.

Regarding TVV strains in *T. vaginalis* isolate G3, our de novo sequencing results and those from the study by Bradic et al. [30] based at New York University (BioProject PRJNA280779) concur in identifying TVV2 and TVV3, but not TVV1, TVV4, or TVV5 in this isolate. Moreover, the high levels of nt sequence identity (≥99.3%) between TVV2-G3(HMS) and TVV2-G3(NYU) and also between TVV3-G3(HMS) and TVV3-G3(NYU) are consistent with the limited divergence of these viruses, although the *T. vaginalis* isolate was cultured first at ATCC and then separately at the two recipient institutions. On the other hand, the discrepancies found for the viruses in this isolate from the study at University of Utah (BioProject PRJNA345042; presence of TVV1, but not TVV2, TVV3, TVV4, or TVV5) are harder to explain, especially given that this isolate was again obtained from ATCC according to BioSample metadata from that study. One possibility would seem to be that the SRA data for isolates G3 and B7RC2 from the University of Utah study might have been transposed in the database, because the current study identified both TVV2 and TVV3 in isolate B7RC2, as expected instead for isolate G3 based on other results. However, TVV2-B7RC2 and TVV3-B7RC2 are substantially divergent from TVV2-G3(HMS) and TVV3-G3(HMS) (≤85.4% nt sequence identity), which makes this explanation seem unlikely. At this stage, then, these discrepancies remain unexplained, but they highlight the need for investigators to confirm the virus content of *T. vaginalis* isolates used in each new study that is focused on these viruses.

The current study also identifies discrepancies in the TVV content of *T. vaginalis* isolate T016. From two studies at Heinrich Heine University Düsseldorf (BioProjects PRJNA176299 and PRJNA236636) [31,33], isolate T016 is found to contain TVV2, but not TVV1, TVV3, TVV4, or TVV5; the TVV2 sequences derived from those two studies are found to be identical. In contrast, isolate T016 from Yonsei University (BioProject PRJNA352855) [32] is found to be negative for all five trichomonasvirus species. In this case, because the discrepancies involve the presence or absence of a single TVV strain, the *T. vaginalis* isolate T016 from Yonsei University might have simply been cured of TVV2 during culture, as has been reported to occur in other cases [47,48].

The double stem–loop structure predicted near the coding-strand 3′ terminus of each analyzed TVV2, TVV3, TVV4, and TVV5 sequence is a striking feature. Conserved secondary structures have been found in other RNA viruses to be involved in replication or packaging. Although this double stem–loop varies in length somewhat between trichomonasviruses, the maintenance of its overall shape in TVV2 through TVV5 suggests a functional role in the viral life cycle. This feature was not found in TVV1, which is not entirely surprising. The evolutionary distance is considerable between TVV1 strains and those of the other four species, as deduced from phylogenetic analyses (see Figure 1) as well as facets of viral biology such as translation strategies. Strains from TVV2 to TVV5 seem to employ a −1 ribosomal frameshifting mechanism for translation of the CP/RdRp fusion protein, whereas TVV1 strains use a −2 frameshifting mechanism. TVV1 may thus possess its own characteristic RNA structures to fulfill vital roles in its life cycle. Future biochemical studies dissecting the functional potential of TVV RNA sequences appear especially warranted.

## Figures and Tables

**Table 1:** Screens of SRA datasets at NCBI for TVV-matching sequence reads.

			Sequence Read Counts from Screen for:				
BioProject	Inst. ^1^	*T. vaginalis* Isolate ^2^	TVV1	TVV2	TVV3	TVV4	TVV5
PRJNA176299	HHUD	T016	0	**18,293 ^3^**	0	0	0
PRJNA236636	HHUD	T016	0	**5254**	0	0	0
PRJNA280779	NYU	BRIS/92/STDL/B7268 ^4^	**1017**	4	0	0	17
PRJNA280779	NYU	GOR/03/PNGIMR/69	**1154**	**2447**	2	0	2
PRJNA280779	NYU	G3	2	**385**	**180**	1	4
PRJNA280779	NYU	NYCA04	**2488**	6	**1029**	2	**2783**
PRJNA280779	NYU	NYCB20	4	6	1	0	4
PRJNA280779	NYU	NYCC37	**2114**	**115**	**117**	3	**519**
PRJNA280779	NYU	NYCD15	**2841**	**4702**	**448**	**348**	**7737**
PRJNA280779	NYU	NYCE32	**694**	3	**119**	**589**	**5539**
PRJNA280779	NYU	NYCF20	**2502**	2	**248**	0	6
PRJNA280779	NYU	NYCG31	**422**	5	**67**	0	**6917**
PRJNA280779	NYU	SD2 11591*	**2727**	0	**166**	0	**133**
PRJNA345042	UU	B7RC2	0	**510**	**238**	0	0
PRJNA345042	UU	G3	**26,085**	4	10	0	0
PRJNA352855	YU	T016	0	0	0	0	0
Current study	HMS	G3	0	**3114**	**10,652**	0	0

**^1^** Institution: HHUD, Heinrich Heine University Düsseldorf; YU, Yonsei University; UU, University of Utah; NYU, New York University; HMS, Harvard Medical School; **^2^** As indicated in the metadata for the respective SRA accessions, including the asterisk in SD2 11591*; **^3^** Numbers in bold reflect new TVV strains; **^4^** SRA reads from this *T. vaginalis* isolate and a metronidazole-resistant mutant derived from it were combined for this analysis.

## Data Availability

RNA-Seq reads were deposited in the SRA database at NCBI under accession code SRX8785706. The bioinformatics code used for analysis in this study has been made freely available in the ‘TVV Transcriptome Mining’ repository at www.github.com/austinreidmanny/tvv-transcriptome-mining (last accessed 27 February 2022).

## Supplementary Materials

The following supporting information can be downloaded at: https://www.mdpi.com/article/10.3390/v14030548/s1, Figure S1: Phylogenetic tree of TVV1 through TVV5 strains based on genome nt sequences. Figure S2: Distribution of TVV assembly lengths versus median coverage depth across each assembly, Figure S3: Normalized mapped reads per trichomonasvirus species. Table S1: Sequence read counts for the new TVV assemblies, Table S2: Sequencing depth values for the new TVV assemblies, Table S3: Primers used for polymerase chain reactions for virus detection in *T. vaginalis* isolate G3. Table S4: Pairwise identity matrix of TVV1 CP/RdRp aa sequences, Table S5: Pairwise identity matrix of TVV2 CP/RdRp aa sequences, Table S6: Pairwise identity matrix of TVV3 CP/RdRp aa sequences, Table S7: Pairwise identity matrix of TVV4 CP/RdRp aa sequences, Table S8: Pairwise identity matrix of TVV5 CP/RdRp aa sequences, File S1: TVV genome nt sequences newly assembled from SRA datasets, File S2: GenBank accession numbers of other sequences used for phylogenetic analyses, File S3: Alignment of CP/RdRp aa sequences used for the phylogenetic analyses represented in Figure 1, File S4: Newick tree file represented in Figure 1 for 100 standard bootstraps, File S5: Newick tree file represented in Figure 1 for 100 standard+transfer bootstraps, File S6: Newick tree file represented in Figure 1 for 1000 ultrafast bootstraps, File S7: Newick tree file represented in Figure 1 for 1000 ultrafast+transfer bootstraps.
