# Supplementary material for: Discovery of a Novel Species of Trichomonasvirus in the Human Parasite Trichomonas vaginalis Using Transcriptome Mining
Source: Viruses. 2022 Mar 6;14(3):548. doi: 10.3390/v14030548 (PMC8953718; doi:10.3390/v14030548)
Supplement: Supplementary file 1 [file viruses-14-00548-s001.zip › viruses-1585377-supplementary.pdf]

viruses

MDPI

Supplementary Materials

Discovery of a New Species of Trichomonasvirus in the Human Parasite *Trichomonas vaginalis* Using Transcriptome Mining

Table S1. Sequence read counts for the new trichomonasvirus assemblies, expressed as RPKM values.

			RPKM values[4] for:				
**BioProject**	**Inst.** [1]	***T. vaginalis* isolate** [2]	**TVV1**	**TVV2**	**TVV3**	**TVV4**	**TVV5**
PRJNA176299	HHUD	T016	na	0.6[5]	na	na	na
PRJNA236636	HHUD	T016	na	4.8[6]	na	na	na
PRJNA280779	NYU	BRIS/92/STDL/B7268 [3]	1.3[6]	na	na	na	na
PRJNA280779	NYU	GOR/03/PNGIMR/69	3.4[6]	7.3[6]	na	na	na
PRJNA280779	NYU	G3	na	1.5[5]	0.6[5]	na	na
PRJNA280779	NYU	NYCA04	6.7[6]	na	2.9[7]	na	11.6[5]
PRJNA280779	NYU	NYCB20	na	na	na	na	na
PRJNA280779	NYU	NYCC37	7.8[6]	0.6[5]	0.5[5]	na	1.7[7]
PRJNA280779	NYU	NYCD15	5.2[6]	8.5[6]	0.8[7]	0.7[5]	12.9[6]
PRJNA280779	NYU	NYCE32	2.0[6]	na	0.4[5]	0.4[5]	14.7[6]
PRJNA280779	NYU	NYCF20	5.0[6]	na	0.6[5]	na	na
PRJNA280779	NYU	NYCG31	1.2[6]	na	0.5[5]	na	0.6[5]
PRJNA280779	NYU	SD2-11591*	7.3[6]	na	0.4[5]	na	0.6[5]
PRJNA345042	UU	B7RC2	na	0.7[7]	0.3[7]	na	na
PRJNA345042	UU	G3	31[6]	na	na	na	na
PRJNA352855	YU	T016	na	na	na	na	na
Current study	HMS	G3	na	56[6]	184[6]	na	na

[1] Institution: HHUD, Heinrich Heine University Düsseldorf; YU, Yonsei University; UU, University of Utah; NYU, New York University; HMS, Harvard Medical School; [2] As indicated in the metadata for the respective SRA accessions, including the asterisk in SD2 11591*; [3] SRA reads from this T. vaginalis isolate and a metronidazole-resistant mutant derived from it were combined for this analysis; [4] Unique assembly-matching sequence reads per (RP) total sequence reads in each respective SRA data set. Values are expressed relative to thousand (K) nt of assembly length and million (M) total sequence reads; [5] Value calculated for the longest partial assembly (contig) obtained for the respective TVV strain; [6] Coding-complete sequence; [7] Nearly coding-complete sequence.

Table S2. Sequencing depth values for the new trichomonasvirus assemblies.

			Median sequencing depth values[4] for:				
**BioProject**	**Inst.** [1]	***T. vaginalis* isolate** [2]	**TVV1**	**TVV2**	**TVV3**	**TVV4**	**TVV5**
PRJNA176299	HHUD	T016	na	7[5]	na	na	na
PRJNA236636	HHUD	T016	na	102[6]	na	na	na
PRJNA280779	NYU	BRIS/92/STDL/B7268 [3]	19[6]	na	na	na	na
PRJNA280779	NYU	GOR/03/PNGIMR/69	20[6]	39[6]	na	na	na
PRJNA280779	NYU	G3	na	10[5]	4[5]	na	na
PRJNA280779	NYU	NYCA04	38[6]	na	16[7]	na	12[5]
PRJNA280779	NYU	NYCB20	na	na	na	na	na
PRJNA280779	NYU	NYCC37	43[6]	3[25]	3[5]	na	7[7]
PRJNA280779	NYU	NYCD15	48[6]	68[6]	7[7]	7[5]	14[6]
PRJNA280779	NYU	NYCE32	13[6]	na	3[5]	3[5]	14[6]
PRJNA280779	NYU	NYCF20	48[6]	na	6[5]	na	na
PRJNA280779	NYU	NYCG31	10[6]	na	3[5]	na	4[5]
PRJNA280779	NYU	SD2-11591*	49[6]	na	3[5]	na	3[5]

PRJNA345042	UU	B7RC2	na	13[7]	6[7]	na	na
PRJNA345042	UU	G3	621[6]	na	na	na	na
PRJNA352855	YU	T016	na	na	na	na	na
Current study	HMS	G3	na	34[6]	161[6]	na	na

[1] Institution: HHUD, Heinrich Heine University Düsseldorf; YU, Yonsei University; UU, University of Utah; NYU, New York University; HMS, Harvard Medical School; [2] As indicated in the metadata for the respective SRA accessions, including the asterisk in SD2 11591*; [3] SRA reads from this T. vaginalis isolate and a metronidazole-resistant mutant derived from it were combined for this analysis; [4] Unique assembly-matching sequence reads overlapping each nt position in the assembly; [5] Value calculated for the longest partial assembly obtained for the respective TVV strain; [6] Coding-complete sequence; [7] Nearly coding-complete sequence.

Table S3. Primers used for polymerase chain reactions for trichomonasvirus detection in *T. vaginalis* isolate G3.

Primer	Sequence
TVV1-G3-F2	CAATACTGTCGCCAGGTGAC
TVV1-G3-R2	GTGTTTGGACAGGTCTTGGAC
TVV2-G3-F1	CGGTTGCTGTGTATTGAGAGG
TVV2-G3-R1	GTCTGTGGTCATCGTAGCG
TVV3-G3-F1	CGCACCTACAATCCAGACG
TVV3-G3-R1	GTGGAAGCGGTTGATGATGG
TVV4-G3-F1	ATGCCAGTTGCTTTCCG
TVV4-G3-R1	TTCCCCAATAGTTATCAG
TVV5-G3-F1	TTCAAGGGTGGTGATCTTCGTAACTG
TVV5-G3-R1	ACTGTACGGTTTGGTAGCCGA

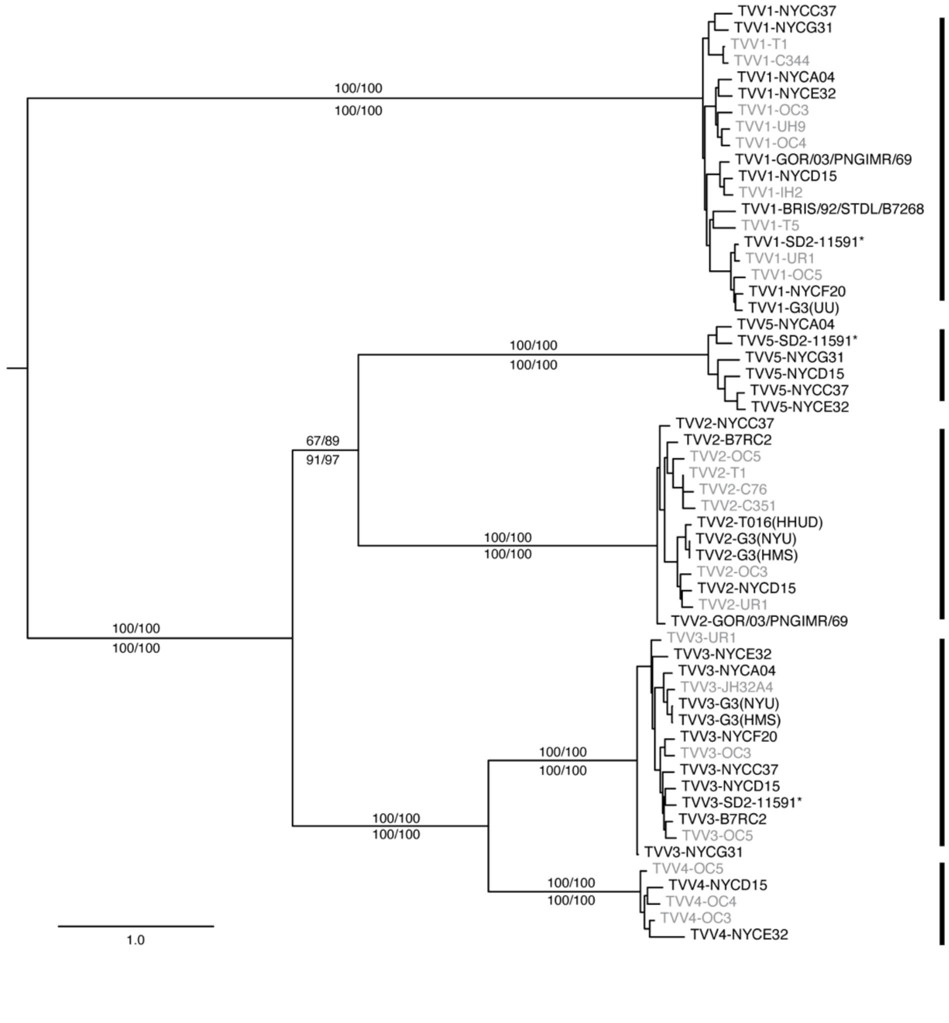

Figure S1. Maximum-likelihood phylogenetic tree of TVV1 through TVV5 strains. Nucleotide (nt) sequences of all new TVV assemblies presented in this study (labeled in black) as well as from reference TVV genomes retrieved from NCBI GenBank (labeled in gray) were used for this analysis. Support values for the main branches are shown as percentages; above the branch is the value from standard bootstrapping without/with subsequent transfer analysis, and below the branch is the value from ultrafast bootstrapping without/with subsequent transfer analysis. The tree is rooted at the midpoint. Bars at right highlight the five trichomonasvirus species. This figure parallels Figure 1 in the main text, which was instead generated using the deduced CP/RdRp aa sequences of these same TVV strains. As in Figure 1, five discrete clades are evident for TVV1 through TVV5, with TVV1 being most divergent and with an apparent sister relationship existing between TVV2 and TVV5 and also between TVV3 and TVV4. The branch uniquely shared by TVV2 and TVV5 has the lowest support values, as also seen in Figure 1, though the support values for this branch are higher here than in Figure 1.

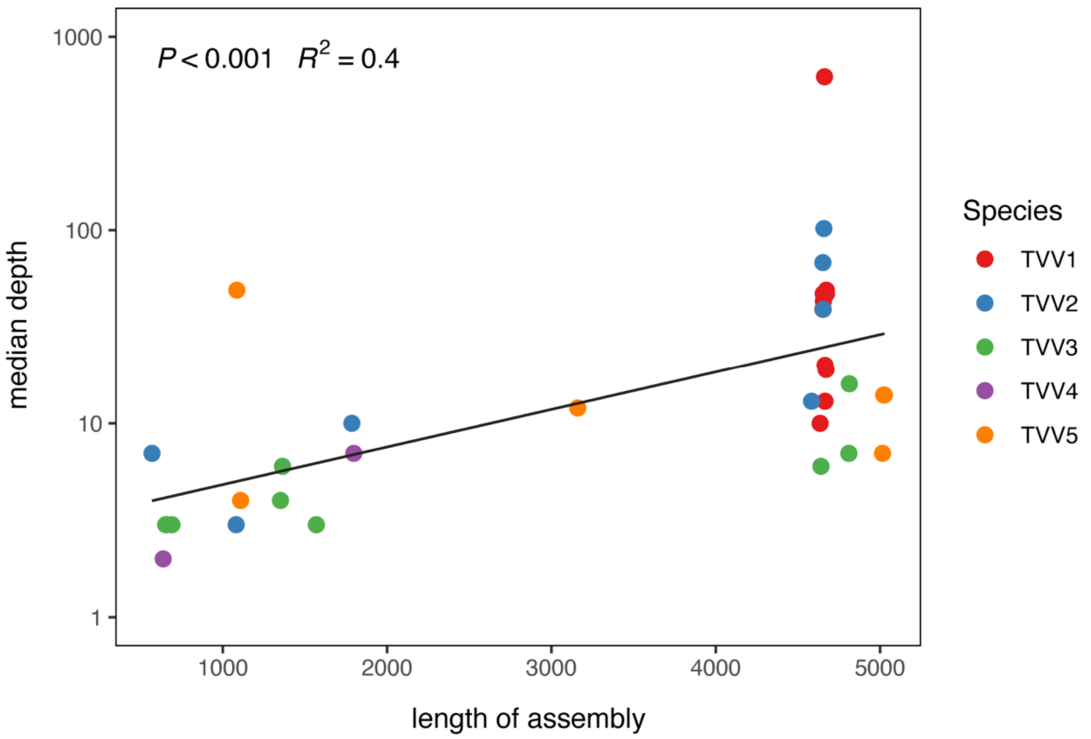

Figure S2. Distribution plot of TVV assembly length (in nt) versus median coverage depth (reads per assembly position) for each new assembly, color-coded per trichomonasvirus species. All new assemblies presented in this study were included in this analysis. The line represents the results of a simple linear regression analysis performed on these data, with the resulting r^2 value and p-value as shown. This analysis was performed to assess whether assembly length and coverage depth are correlated. The regression analysis indeed supports this correlation, with higher coverage depths favoring longer (coding-complete or nearly so) assemblies. The fact that many TVV genomes exhibited low coverage depths is likely explained by the fact that these RNA-Seq datasets represent metatranscriptomes from *T. vaginalis* samples not enriched for TVV RNAs.

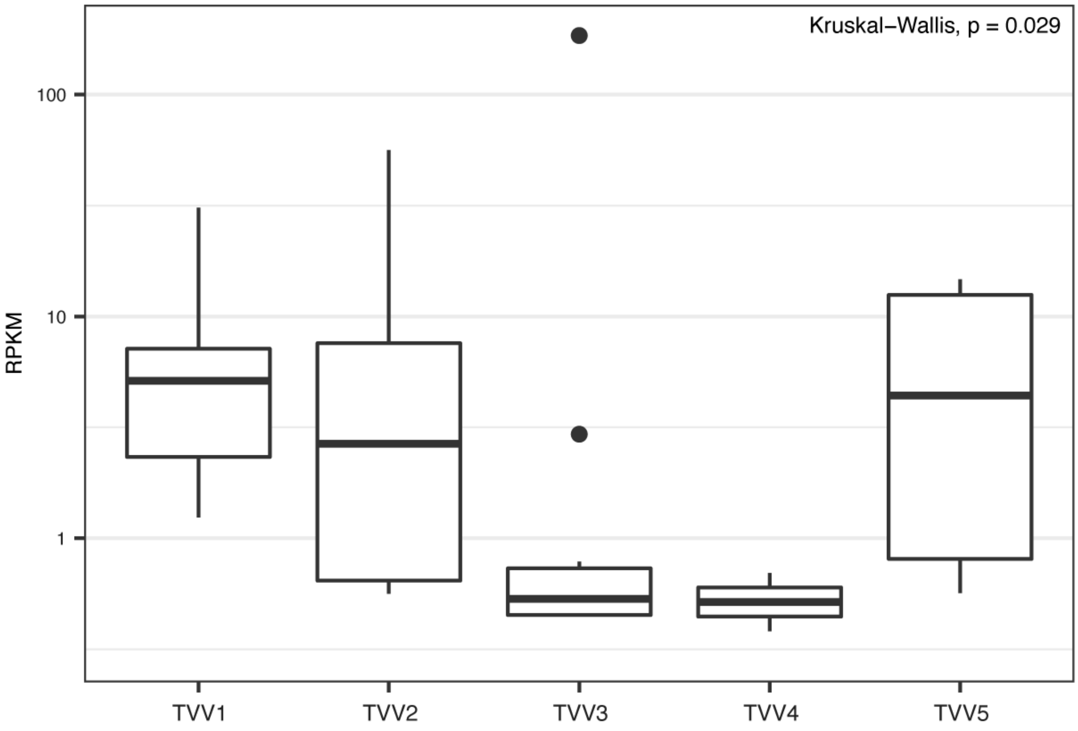

Figure S3. Box-and-whisker plots of mapped reads, normalized by RPKM), per trichomonasvirus species. All new assemblies presented in this study were included in this analysis. The median RPKM value for each species is presented as a bold horizontal line. Upper and lower quartiles are represented respectively by the top and bottom of each box, and upper and lower extremes are indicated by the whiskers (vertical lines). Two outliers interpreted to be present in the values for TVV3 are shown as black dots. A nonparametric Kruskal-Wallis one-way ANOVA test was performed, with the resulting *p*-value as shown. This test was used to assess whether the abundance of viral reads was equivalent between species. Instead, the numbers of TVV-matching reads were found to vary across species, suggesting different levels of transcription or replication for the different species. The fact that many of the TVV genomes exhibited low RPKM values is likely explained by the fact that these RNA-Seq datasets represent metatranscriptomes from *T. vaginalis* samples not enriched for TVV RNAs.
